# Lucius phenomenon: the importance of a primary dermatological care^☆^

**DOI:** 10.1016/j.abd.2020.08.033

**Published:** 2021-11-19

**Authors:** Juliana Viana Pinheiro, Maria Araci de Andrade Pontes, José Urbano de Medeiros Neto, Heitor de Sá Gonçalves

**Affiliations:** Centro de Referência Nacional em Dermatologia Sanitária Dona Libânia, Fortaleza, CE, Brazil

**Keywords:** Early diagnosis, Leprosy, Necrosis

## Abstract

Leprosy presents a varied clinical spectrum. Lucius phenomenon is a rare leprosy reaction characterized by erythematous, painful, slightly infiltrated macules and hemorrhagic bullae that progress to ulceration. This case report describes a patient whose diagnosis of leprosy occurred in the presence of Lucius phenomenon. Late diagnosis and delay in the implementation of specific therapy contributed to an unfavorable outcome, highlighting the importance of early identification and treatment of this disease, as well as its complications.

## Introduction

Leprosy is transmitted by *Mycobacterium leprae*. The clinical picture and reaction states are influenced by the individual's immune response.^1^, ^2^ There are two well-described forms of the reactions: type 1 and type 2.^3^

Lucius phenomenon (LP) is a rare, severe, and diffuse manifestation. Difficulty in the classification of this condition has been going on for years.^4^ Some authors consider it a variant of the type 2 reaction and others as a third reaction pattern, associated with a coagulation disorder.^1^, ^3^

Brazil persists with high incidence rates of leprosy. Between 2014 and 2018, the average detection rate was 13.64 new cases for every 100,000 inhabitants. The high rate of endemicity reinforces the importance of establishing management measures to attain early detection, minimizing deformities and physical disabilities.^5^

The authors describe a case of lepromatous leprosy (LL) that was diagnosed in the presence of LP, despite having been previously assisted at other health services, showing an unfavorable outcome.

## Case report

A 69-year-old man presented with an infectious condition in his left leg for 15 days, associated with fever and chills. He had received amoxicillin and clavulanate, after an evaluation at a Basic Health Unit. He developed painless polygonal blisters, with spontaneous rupture and formation of ulcerated plaques with a necrotic background, initially on the lower limbs, with an ascending pattern. He sought care at an Emergency Unit, where treatment was initiated with meropenem, followed by ceftriaxone associated with clindamycin.

He was admitted to the general ward of a referral hospital for contagious infectious diseases with a diagnostic hypothesis of pharmacoderma, with impaired general status and fever. He reported reduced tactile sensitivity, predominantly on the limbs. Then, the antibiotic regimen was suspended, and prednisone was introduced at a dose of 40 mg/day, and a dermatology assessment was requested.

**Figure 1:**
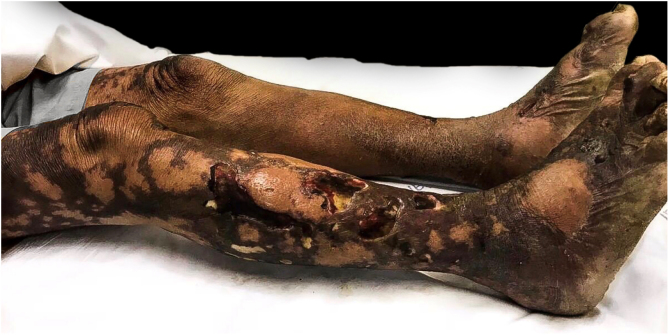
Ulcerated plaques with necrosis and presence of fibrin.

**Figure 2:**
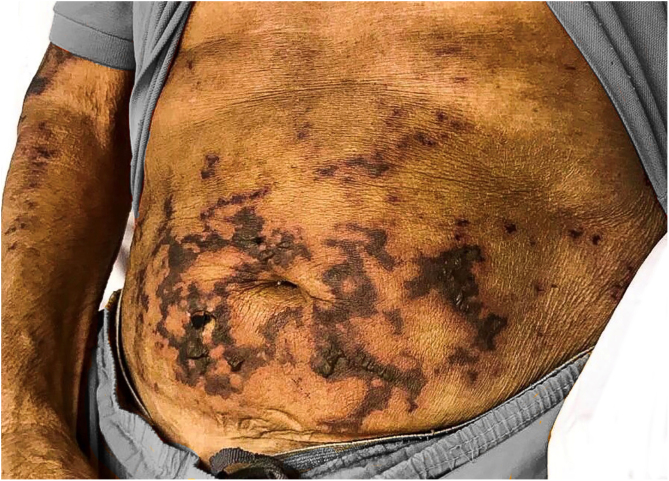
Necrotic areas on the abdomen.

**Figure 3:**
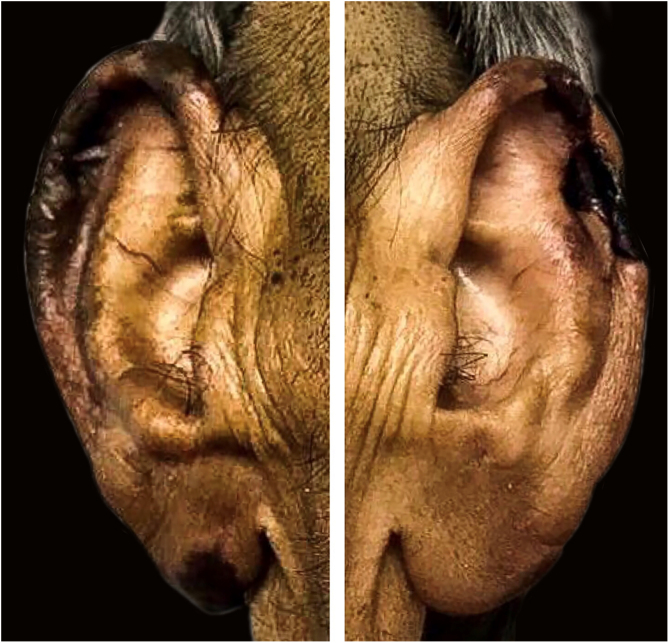
Necrosis on the ear pinnae.

On physical examination, there were ulcerated plaques with a necrotic background, with the presence of fibrin and purulent secretion, affecting the lower limbs, abdomen, buttocks, upper limbs, ear pinnae, and upper lip, bilateral palpable and painful lymph nodes in the inguinal regions and scrotal sac edema (Figure 1, Figure 2, Figure 3). Bilateral madarosis and burn injuries on the fingers were important findings, which raised the hypothesis of leprosy, and bacilloscopy of a dermal infiltrate was requested, which showed a bacilloscopic index of 5. Once the diagnosis was confirmed, standard polychemotherapy for lepromatous leprosy was started.

**Figure 4:**
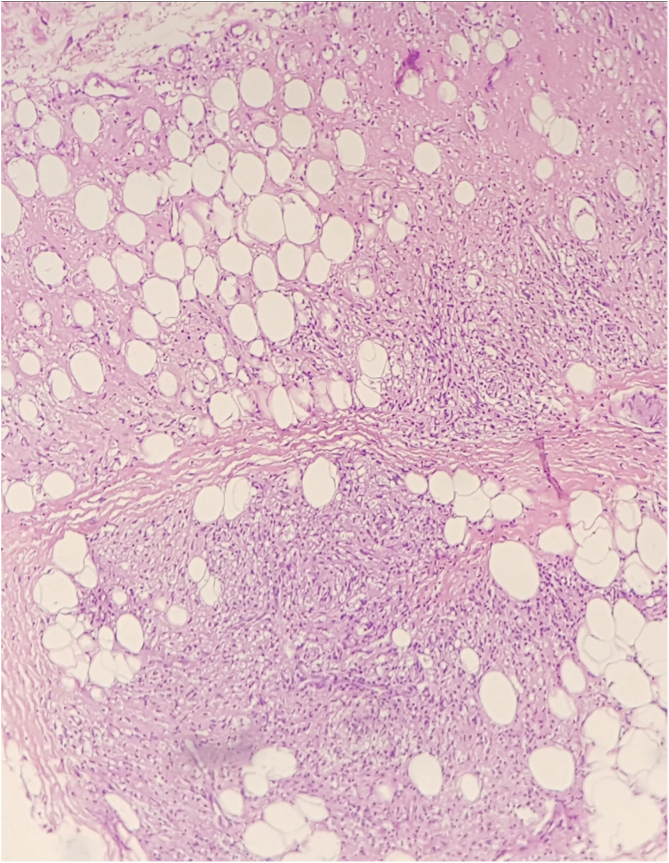
Histological section stained with Hematoxylin & eosin showing a granulomatous inflammatory infiltrate in the subcutaneous adipose tissue.

**Figure 5:**
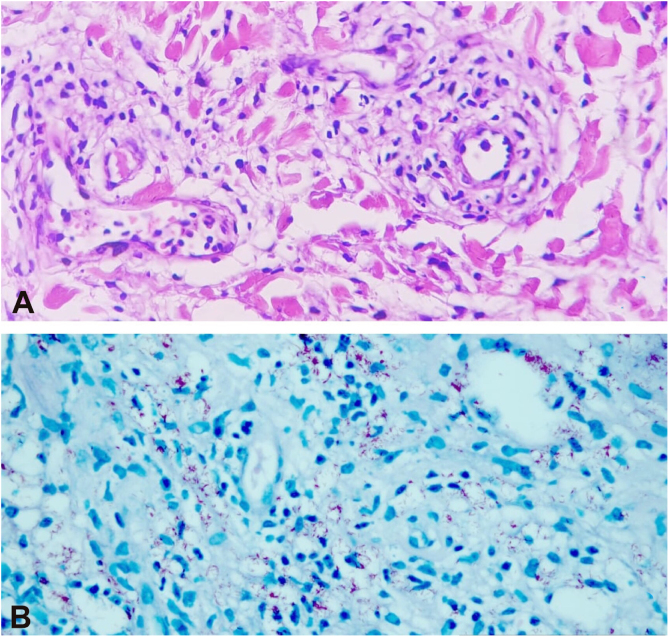
(A), Histological sections showing a neutrophilic infiltrate inside and around the vessels and discrete fibrin deposits (Hematoxylin & eosin, ×400). (B), Numerous acid-fast bacilli (Ziehl-Neelsen, ×400).

The histopathological examination of the biopsies performed at two sites showed an extensive dermal infiltrate predominantly consisting of interstitial, superficial and deep foamy macrophages, full of bacilli, forming globia, in addition to neutrophils around and inside the vessel walls and presence of bacilli in the vessel lumen, leukocytoclasia and extravasation of red blood cells, characterizing leukocytoclastic vasculitis, compatible with LP (Figure 4, Figure 5)

After the histopathological examination, the dose of prednisone was adjusted to 1 mg/kg/day and thalidomide 100 mg/day was introduced.

Despite repeated surgical debridement to remove devitalized tissues and the use of broad-spectrum antibiotics such as vancomycin and piperacillin/tazobactam, the lesions persisted with the secretion of purulent-fibrinous material, suggestive of secondary infection.

The patient developed persistent fever and worsening of the general status. Because he had a recurrent pleural effusion, he was submitted to bronchoscopy, with alveolar bronchial lavage analysis, wich revealed a weakly positive GeneXpert. The RIPE regimen was prescribed; however, the patient died of probable sepsis due to a cutaneous focus on the third day.

## Discussion

The diagnosis of leprosy is essentially clinical and should be preferably performed during primary case assistance. An early diagnosis prevents sequelae and interrupts the transmission chain. However, the varied clinical spectrum of the disease, depending on the individual immune response, makes diagnosis difficult by non-dermatologists.^1^, ^3^, ^6^

LP is a reaction condition characterized by outbreaks of erythematous, painful, slightly infiltrated macules, and hemorrhagic bullae that progress to ulceration. Several factors can precipitate it, such as infections, drugs, and pregnancy.^2^, ^4^ It usually tends to progress with the formation of atrophic stellar scars.^4^, ^7^

It usually appears three to four years after disease onset in untreated or inadequately treated patients.^3^, ^4^ The evolution pattern usually starts in the lower limbs, ascending to the buttocks, upper limbs, hands, and rarely the back and face.^3^ A triad of diagnostic criteria define LP: skin ulceration, vascular thrombosis, and invasion of the blood vessel wall by Hansen's bacilli.^7^

The pathophysiology of occlusive thrombi is yet to be completely elucidated, and it may be due to two mechanisms, one of which is the result of immune-mediated events, whereas the other is a direct effect of the presence of *Mycobacterium leprae* itself in the vessel.^3^ Ischemia, infarction, and tissue necrosis can occur as a result of these events, including disseminated intravascular coagulation.^7^

There have been reports of gastrointestinal alterations in the presence of LP, with the presence of necrotizing and ulcerative lesions in the digestive tract.^8^ Therefore, the parenteral administration of corticoids is recommended. A controversial issue is recommending the use of thalidomide in cases in which LP occurs together with erythema nodosum leprosum (ENL). However, there is no consensus in the literature regarding the use or not of thalidomide in patients without previous ENL.^2^, ^4^, ^7^

The presence of pleural effusions in LP patients has been confirmed in autopsies.^3^, ^9^ GeneXpert detects the rpoB gene more specifically for *M. tuberculosis*; however, weakly positive samples can occur in cases of leprosy with a high bacillary index. A study by the Federal Drug Administration lists *M. leprae* as potentially causing a GeneXpert cross-reaction.^10^ Therefore, it is questionnable whether the pleural effusion, in this case, is also a manifestation of LP, or a co-infection with other mycobacteria.

This case report describes an undiagnosed LL patient, which initially went untreated and that evolved with LP. The unfavorable outcome described herein is relevant, as it reinforces the need for continuing education of professionals working at all levels of healthy care, aiming at attaining an early diagnosis of leprosy and immediate access to adequate treatment, as well as the management of complications. Likewise, the importance of the dermatologists in the care team for treating leprosy and other dermatoses in the hospital environment is emphasized.

## Financial support

None declared.

## Authors' contributions

Juliana Viana Pinheiro: Drafting and editing of the manuscript; intellectual participation in propaedeutic and/or therapeutic conduct of the studied cases; critical review of the literature; critical review of the manuscript.

Maria Araci de Andrade Pontes: Approval of the final version of the manuscript; critical review of the literature; critical review of the manuscript.

José Urbano de Medeiros Neto: Approval of the final version of the manuscript; collection, analysis, and interpretation of data.

Heitor de Sá Gonçalves: Approval of the final version of the manuscript; effective participation in research orientation; critical review of the literature; critical review of the manuscript.

## Conflicts of interest

None declared.
